# Correlation and Influencing Factor Analysis Between Gal-3 and Recurrent Patients With Nonvalvular Atrial Fibrillation After Radiofrequency Ablation

**DOI:** 10.1155/cdr/5561883

**Published:** 2025-08-13

**Authors:** Jing Lu, Fan Yang, Yu Gao, Ruijuan Li, Runqin Li, Nan Zhang, Yan Ren, Dengfeng Ma

**Affiliations:** ^1^Department of Cardiology of Taiyuan Central Hospital, Taiyuan, Shanxi Province, China; ^2^Department of Pharmacy, Shanxi Bethune Hospital, Shanxi Academy of Medical Sciences, Tongji Shanxi Hospital, Third Hospital of Shanxi Medical University, Taiyuan, China; ^3^Tongji Hospital, Tongji Medical College, Huazhong University of Science and Technology, Wuhan, China

**Keywords:** AF ablation, atrial fibrillation, catheter ablation, fibrosis, galectin-3, recurrence

## Abstract

**Aims:** This prospective cohort study was aimed at evaluating the association of galectin-3 (Gal-3) and other prognostic factors with atrial fibrillation (AF) recurrence in patients with nonvalvular AF undergoing radiofrequency catheter ablation (RFCA).

**Methods and Results:** Overall, 92 patients were included, with the AF group comprising 70 patients and the control group comprising 20 (supraventricular tachycardic) patients. Preablation parameters were recorded, including baseline Gal-3 levels. Patients were followed up for 6 months post-RFCA. AF recurrence was defined as AF episodes lasting > 30 s after the blanking period. Univariate and multivariate logistic regression analyses revealed patients with AF were older and significantly hypertensive (*p* < 0.05) when compared to control. Gal-3 levels were significantly higher in the paAF (13.81 ± 2.56) and peAF (15.92 ± 3.67) groups than in the control group (12.04 ± 3.28). Recurrence occurred in 28 patients (40%) with AF. Baseline Gal-3 levels were higher in the recurrence group than in the nonrecurrence group. Univariate analysis revealed left atrial diameter (LAD), left atrial volume (LAV), high-sensitivity C-reactive protein (hs-CRP), Gal-3, and LV short-axis shortening rate as significant predictors of AF recurrence. However, multivariate analyses showed only LAV (OR = 1.043 [1.011–1.077]; *p* = 0.009), hs-CRP (OR = 1.176 [1.025–1.349], *p* = 0.021), and Gal-3 (OR = 2.019 [1.332–3.06], *p* = 0.001) as independent predictors of recurrence.

**Conclusion:** Elevated Gal-3 and hs-CRP levels along with increased LAV are predictive of AF recurrence, irrespective of AF type. This finding holds a significant potential for guiding therapeutic strategies in clinical practice.

## 1. Introduction

Atrial fibrillation (AF) is a common cardiac arrhythmia and a significant public health concern [1]. It imposes substantial burdens in terms of disability, mortality, and diminished quality of life [2, 3]. Individuals with AF face a higher risk of heart failure, stroke, and all-cause mortality than those without AF [2, 4]. Globally, AF is estimated to impact 2%–4% of the adult population, with projections suggesting a prevalence of approximately 16 million by 2050 [5, 6]. In China, the prevalence of AF among adults is 1.6%, showing significant regional differences. Key factors contributing to AF include older age, male sex, and underlying cardiovascular disease [6].

Catheter ablation (CA), including radiofrequency ablation and cryoablation, has become an alternative therapeutic option for symptomatic AF patients who are unresponsive to antiarrhythmic drugs. Radiofrequency catheter ablation (RFCA) is crucial for isolating pulmonary veins and preventing AF initiation [3, 4]. Predicting the efficacy of RFCA on an individual basis remains challenging. Previous studies have reported varying success rates, typically ranging from 50% to 80% [3, 4, 7]. Notably, around one-third of patients undergoing the procedure may encounter AF recurrence, even after multiple attempts. The recurrence rate may increase to 50% in individuals with persistent atrial fibrillation (peAF) [2, 8]. The 5-year recurrence rate of AF after a single ablation procedure is reported to be up to 70% [5].

Several risk factors contributing to the progression of AF have been identified [9]. Atrial remodeling, characterized by fibrosis, disrupts electromechanical conduction and fosters an environment conducive to AF [4, 9]. Galectin-3 (Gal-3) has emerged as a significant biomarker in cardiac pathology due to its involvement in cardiac fibrosis, inflammation, and remodeling processes. Recent studies have revealed an association between circulating Gal-3 levels and AF recurrence [1, 3, 4, 8, 10–14]. Nevertheless, the evidence concerning AF has been contentious, as certain studies have highlighted prognostic significance while others have shown limited utility [11]. Additionally, other biomarkers such as natriuretic peptides, C-reactive protein (CRP), interleukin-6 (IL-6), low-density lipoprotein (LDL), and tissue inhibitor of metalloproteinase-2 (TIMP) have also been associated. Furthermore, factors such as left atrial diameter (LAD) and left atrial volume (LAV) have been extensively documented as predictors of AF recurrence [2, 5, 15].

Enhancing the comprehension of baseline clinical indicators predicting AF recurrence is vital for managing patients with AF after RFCA. It has the potential to pave the way for personalized medicine, enabling the risk stratification of patients before invasive interventions or the identification of novel drug targets. Hence, the present study evaluated the association between serum Gal-3 levels and other prognostic factors for AF recurrence after RFCA. The objectives were to (a) evaluate and compare Gal-3 levels between different types of AF and the control group and (b) evaluate the prognostic clinical factors for recurrence after RFCA in patients with nonvalvular AF.

## 2. Results

### 2.1. Baseline Characteristics

The baseline characteristics of the study population are shown in Table 1. Most patients in all groups were male (*p* > 0.05). However, the three groups showed significant differences in age, BMI, and comorbidities such as diabetes mellitus (DM), lipid metabolism disorder, obstructive sleep apnea/hypopnea syndrome (OSAHS), and cerebrovascular events. Compared to the control group, patients with AF tended to be older and more hypertensive (*p* < 0.05).

### 2.2. Blood Samples and Gal-3 Measurements

Table 2 compares inter- and intragroup echocardiographic and laboratory parameters. An overall significant difference was observed for all echocardiographic factors among the three groups, while differences were noted for hematocrit (HCT), brain natriuretic peptide (BNP), and Gal-3 among laboratory parameters. Gal-3 levels were significantly higher in the paroxysmal atrial fibrillation (paAF) (13.81 ± 2.56) and peAF (15.92 ± 3.67) groups compared to the control group (12.04 ± 3.28). Furthermore, Gal-3 levels were significantly higher in the peAF group than in the paAF group (*p* < 0.05).

### 2.3. RFCA Procedure and Follow-Up

No patient was lost to follow-up after 6 months. There were 28 (40%) cases of AF recurrence after RFCA, including seven cases with paAF and 11 cases with peAF at the end of the blanking period.

At the 6-month follow-up after RFCA, a total of 28 patients (40%) experienced AF recurrence. Of these, 18 cases (seven with paAF and 11 with peAF) were identified at the end of the 90-day blanking period, while the remaining 10 patients had late recurrences between 3 and 6 months postprocedure.

A comparison between the recurrence and nonrecurrence groups revealed significant differences in LAD, LAV, high-sensitivity C-reactive protein (hs-CRP), Gal-3, and LV short-axis shortening rate (*p* < 0.05) (Table 3). Logistic regression analysis evaluated the effects of the significant variables identified in the univariate analysis on AF recurrence. In multivariate logistic regression with recurrence as the dependent variable, LAV (odds ratio [OR] = 1.043, 95%confidence interval [CI] = 1.011‐1.077; *p* = 0.009), hs-CRP (OR = 1.176, 95%CI = 1.025‐1.349, *p* = 0.021), and Gal-3 (OR = 2.019, 95%CI = 1.332‐3.06, *p* = 0.001) were significantly related (Table 4).

## 3. Discussion

The results of this study indicate that LAV, hs-CRP, and Gal-3 hold clinical significance in predicting AF recurrence, thereby assisting in a minimally invasive method of improved patient selection. These findings are consistent with those in the existing literature. The study cohort represents a “real-world” population with strict exclusion criteria, thus enhancing the generalizability of the findings. Despite advances in the therapeutic strategies in patients with AF, recurrence remains a significant challenge. Better risk stratification of candidates for rhythm control therapy is imperative. Identifying predictors of AF recurrence is essential for guiding post-RFCA patient management, such as determining the duration of antiarrhythmic drug therapy, monitoring for asymptomatic recurrence, and establishing realistic patient expectations [5]. Hence, this study was aimed at ascertaining the predictors of recurrence by analyzing clinical and echocardiographic parameters.

This study observed recurrence in 28 patients (40%) with AF at 6 months follow-up. The literature varied on the follow-up period. Ruan et al. reported 22.88% recurrence (6 months follow-up), and Clementy et al. reported 34% recurrence (1-year follow-up) [10, 13]. A previous meta-analysis by Gong et al. supports the finding that higher Gal-3 levels were in the peAF (15.92 ± 3.67) group than in the paAF (13.81 ± 2.56) group [11].

Although the causative relationship between LA enlargement and AF remains uncertain, substantial evidence suggests that a larger LA size perpetuates the vicious cycle of atrial remodeling and AF. Furthermore, structural remodeling, atrial hypertrophy, and stretching can induce ionic currents and changes in electrical remodeling [5]. Additionally, the LA is subjected to elevated LV filling pressures during diastole. Prolonged exposure to these elevated pressures leads to LA remodeling and stiffening, manifesting as enlargement and increased blood volume [2]. A meta-analysis involving 22 studies demonstrated a correlation between increased anteroposterior diameter of the LA and heightened AF recurrence post-RFCA [16]. However, this current study did not find a significant predictive effect of LAD on AF recurrence. The significant association of LAV with AF recurrence in this study is consistent with the findings of the Shin et al. study [15]. Meta-analyses of 13 studies involving 2886 subjects indicated that larger LAV/LAVi independently correlated with notably higher odds of AF recurrence (OR = 1.032, 95%CI = 1.012‐1.052; *p* = 0.001) [5].

hs-CRP is a widely used inflammatory marker that is capable of predicting AF recurrence [17]. In this study, for every unit change in hs-CRP, there is a 17% higher risk of AF recurrence (OR = 1.176, 95%CI = 1.025‐1.349, *p* = 0.021). This result aligns with the existing literature. A 2013 meta-analysis encompassing seven studies revealed a significant positive correlation between elevated baseline hs-CRP concentration and AF recurrence [18]. Based on 21 studies (5049 patients), pooled standardized mean difference for baseline hs-CRP showed that levels were higher in the recurrence group than in the nonrecurrence group postablation (OR = 2.04, 95% CI: 1.28–3.23, *p* < 0.01) [1]. Among 789 patients from 10 studies conducted after AF ablation, the mean hs-CRP level was higher in the recurrent group (0.72 ± 0.73 mg/dL) than in the nonrecurrent group (0.38 ± 0.51 mg/dL) [17].

Patients who experienced AF recurrence postablation exhibited significantly higher baseline Gal-3 levels than those who did not. This finding is consistent with previous studies (Table 5) [3, 8, 10–12, 14, 19]. In this study, each incremental increase of 1 ng/mL in Gal-3 is associated with a 2.01-fold increase in recurrence odds (OR = 2.019, 95%CI = 1.332‐3.06; *p* = 0.001). Lee et al. reported a hazard ratio of 1.39 (CI = 1.14‐1.70, *p* = 0.001) [3]. A meta-analysis from 2019 indicated that a 1 ng/mL elevation in baseline Gal-3 corresponded to a 17% higher risk of AF recurrence, independent of patient age, gender, and baseline LAD (RR = 1.17, 95%CI = 1.01‐1.35; *p* = 0.03) [4]. Another recent meta-analysis found that serum Gal-3 was linked to an increased risk of AF recurrence (HR = 1.25, 95%CI = 1.01‐1.55; *p* = 0.04) [20]. A comparison with individual studies is shown in Table 5.

The present study has several limitations. Comorbidities like hypertension, DM, and coronary heart disease (CHD) may affect myocardial fibrosis and remodeling, potentially causing inaccuracies in Gal-3 serum level measurements. The blood sample was taken from a peripheral vein instead of directly from atrial tissue, possibly indicating other fibrotic or inflammatory conditions. Asymptomatic episodes of AF might not have been detected during the follow-up period. The study population size was relatively small because of the strict enrollment criteria. This was a single-center study with a small number of patients and a short follow-up duration.

### 3.1. Limitations

This study has several limitations. The relatively small, single-center cohort and short follow-up duration may limit the statistical power and generalizability of the findings. Although strict inclusion criteria were used to minimize confounding, they also reduced the sample size. Comorbidities such as hypertension, diabetes, and coronary artery disease may have influenced Gal-3 levels through mechanisms unrelated to atrial remodeling. Additionally, Gal-3 was measured from peripheral venous blood rather than atrial tissue, which may reflect systemic rather than cardiac-specific fibrosis or inflammation. The absence of postrecurrence Gal-3 measurements and the possibility of undetected asymptomatic AF episodes further limit the interpretation of recurrence outcomes. Finally, detailed atrial substrate characterization and ablation variability were not systematically assessed.

In conclusion, higher levels of Gal-3 and hs-CRP and increased LAV predict AF recurrence, regardless of AF type. This finding holds significant potential for guiding therapeutic strategies in clinical practice, enabling the personalization of pharmacological and interventional AF therapies. Nonetheless, further studies, mainly focusing on establishing cutoff points, are imperative before their integration into routine risk stratification for AF ablation.

## 4. Methods

### 4.1. Study Design and Ethical Considerations

This prospective cohort study was conducted from January 2022 to July 2023 at Taiyuan Central Hospital. The study was approved by the Medical Research Ethics Committee of Taiyuan Central Hospital (Approval Number 2023022). The protocol was consistent with the ethical guidelines of the 2008 Declaration of Helsinki. Written informed consent was obtained from all participants.

### 4.2. Study Population

A total of 92 consecutive participants who met the criteria of 2020 ESC diagnostic and management guidelines and underwent RFCA were enrolled in the study [21]. The AF group consisted of 70 patients (37 with paAF and 33 with peAF). Twenty-two patients with supraventricular tachycardia were included in the control group. All methods, including the RFCA procedure, were performed according to the guidelines [22].

Participants were excluded if their (a) age was less than 18 years; (b) had secondary to rheumatic heart disease, primary cardiomyopathy, congenital heart disease, infectious endocarditis, and other secondary AF; (c) concomitant fibrosis-related diseases such as pulmonary fibrosis and cirrhosis; (d) suffering from mental illness; and (e) opted for cardiac surgery.

### 4.3. Preoperative Data Collection

Sociodemographic parameters (age and sex), altered habits (smoking history and drinking history), comorbidities (DM, hypertension, lipid metabolism disorder, CHD, OSAHS, and cerebrovascular events) were recorded. All patients underwent routine preoperative echocardiographic and laboratory evaluation.

Transthoracic echocardiography, cardiac magnetic resonance imaging, or computed tomography scans were used for baseline assessments before RFCA. These assessments included parameters such as LAD, end-diastolic left ventricular diameter (LVDD), left ventricular short-axis shortening rate, and left ventricular ejection fraction (LVEF). Additionally, to rule out the presence of an intra-atrial thrombus, transesophageal echocardiography or dual-source coronary CT angiography was performed.

### 4.4. Blood Samples and Gal-3 Measurements

Before RFCA, blood was aspirated from the peripheral vein for biomarker and parameter analysis. The aspirated blood was then transferred to serum separator tubes and left to clot for a minimum of 30 min. After clot formation, the tubes were centrifuged at 1600 g for 15 min. Aliquots of the separated serum were transferred to sterile, nonpyrogenic Eppendorf tubes and stored at −70°C until analysis, undergoing only one freeze-thaw cycle before analysis. Fibrosis-related biomarkers were assessed using commercially available enzyme-linked immunosorbent assay (ELISA) kits. Quantitative analysis of plasma Gal-3 concentrations and BNP levels was performed using kits from Wuhan Huamei Biotechnology, following the manufacturer's instructions. All samples were assessed in duplicate, and the values were normalized to a standard curve, with the lower detection limit set at 0.005 ng/mL. Additionally, HCT, hs-CRP, triglyceride (TG), and uric acid (UA) levels were concurrently evaluated.

### 4.5. RFCA Procedure

Access to the right and left femoral veins was obtained under either general anesthesia or conscious sedation with a local anesthetic. Intravenous heparin was administered to ensure an activated clotting time > 300 s. Following the transseptal puncture, regardless of the patient's rhythm, bipolar voltages of the LA were captured using a high-density multielectrode circular EP mapping catheter and a 3D mapping system (CARTO R3; Biosense Webster, Irvine, CA, United States). The mapping catheter was systematically maneuvered across the entire LA endocardium, with each electrode position recorded for at least 2 s to accommodate potential voltage variations over time, particularly in patients with AF during mapping. The mean pressures of the right and LA were evaluated by transducing the LA sheath before and after the transseptal puncture.

Radiofrequency energy was employed to perform wide-area circumferential ablation to achieve pulmonary vein isolation (PVI), utilizing a contact force-sensing ablation catheter. In non-paAF cases, linear ablation and ablation of complex fractionated electrograms were performed based on the operator's judgment. Successful PVI was confirmed in all patients by demonstrating the exit and entry blocks. Repeat ablation was performed if the patient had symptomatic arrhythmia recurrence after the blanking period and the patient and clinician felt that these ongoing symptoms justified further intervention. During repeat procedures, PVI was reassessed, and if veins were reconnected, targeted ablation was performed to restore isolation. LA substrate mapping and comprehensive ablation of low-voltage areas were performed for peAF. Potentials with amplitudes exceeding 0.5 mV were classified as normal, while those below 0.2 mV were designated as low voltage.

### 4.6. Follow-Up

All patients were prescribed antiarrhythmic drugs unless they had contraindications or experienced intolerance. Medications were discontinued if no recurrent atrial tachyarrhythmia was observed 2–3 months after RFCA. Warfarin was administered to all patients for at least 3 months following the procedure, along with proton pump inhibitors added for 4 weeks. Ambulatory monitoring and 12-lead electrocardiography were performed 1–2 days after the procedure during the patient's hospital stay. An electrocardiography was routinely performed at each visit in the outpatient clinic. The planned follow-up duration was 6 months. At 3 and 6 months, a 24-h Holter monitoring was conducted with clinical examinations and resting electrocardiograms (ECGs).

The 3 months following radiofrequency ablation was termed the “blank period.” The primary endpoint was arrhythmia recurrence after the 90-day blanking period. The recurrence of arrhythmia was defined as symptomatic or asymptomatic AF or atrial tachycardia lasting longer than 30 s, diagnosed via a 12-lead ECG or Holter monitor following the 3-month blanking period. Any instance of AF, atrial flutter, or atrial tachycardia during this timeframe was categorized as early AF recurrence. The emergence of AF between 3 and 12 months postsurgery was termed late recurrence.

### 4.7. Statistical Analysis

Statistical analyses were performed using IBM SPSS Statistics for Windows, Version 25.0. Armonk, NY: IBM Corp. Continuous measurement results are presented as mean ± standard deviation, median (first quartile and third quartile), and categorical results as frequency (percentage). The normality of the data was assessed using the Shapiro–Wilk test. Continuous variables were compared between the two groups using an independent sample *t*-test/Mann–Whitney rank sum test and categorical variables using the chi-square test/Fisher's exact probability test. Analysis of variance and Fisher's least significant difference multiple comparisons were used to compare multiple groups. Factors that were significant in the univariate analysis were considered in the multivariate logistic regression analysis. Statistical significance was set at *p* value < 0.05.

## Figures and Tables

**Table 1:** Comparison of baseline characteristics.

**Variables**	**PSVT (** **n** = 22**)**	**paAF (** **n** = 37**)**	**peAF (** **n** = 33**)**	**χ** ^2^/**F**	**p** ** value**
Males, *n* (%)	12 (54.5)	22 (59.5)	26 (78.8)	4.324	0.115
Mean age (years)	53.95 ± 14.34	66.46 ± 9.39^∗^	64.88 ± 7.56^∗^	11.264	< 0.001
Mean BMI (kg/m^2^)	24.28 ± 3.25	24.90 ± 3.49	26.46 ± 3.30^∗^	3.210	0.045
Mean SBP (mm Hg)	129.50 ± 22.11	138.62 ± 22.72	128.00 ± 15.83	2.718	0.072
Mean DBP (mm Hg)	82.64 ± 15.11	84.30 ± 10.88	85.58 ± 11.60	0.381	0.684
Smoking history, *n* (%)	10 (45.5)	11 (29.7)	18 (54.5)	4.510	0.105
Drinking history, *n* (%)	8 (36.4)	8 (21.6)	11 (33.3)	1.840	0.398
Hypertension, *n* (%)	6 (27.3)	23 (62.2)⁣^∗^	23 (69.7)⁣^∗^	10.469	0.005
DM, *n* (%)	5 (22.7)	2 (5.4)	4 (12.1)		0.156^a^
Lipid metabolism disorder, *n* (%)	4 (18.2)	2 (5.4)	6 (18.2)		0.179^a^
CHD, *n* (%)	5 (22.7)	20 (54.1)	14 (42.4)	5.544	0.063
OSAHS, *n* (%)	1 (4.5)	1 (2.7)	3 (9.1)		0.533^a^
Cerebrovascular events, *n* (%)	2 (9.1)	1 (2.7)	2 (6.1)		0.625^a^

*Note:* The parameters not marked with chi-square values in the table were analyzed using Fisher's exact probability method.

Abbreviations: BMI, body mass index; CHD, coronary heart disease; DBP, diastolic blood pressure; DM, diabetes mellitus; OSAHS, obstructive sleep apnea/hypopnea syndrome; paAF, paroxysmal atrial fibrillation; peAF, persistent atrial fibrillation; PSVT, paroxysmal supraventricular tachycardia; SBP, systolic blood pressure.

^a^Analyzed using Fisher's exact probability method.

⁣^∗^*p* < 0.05 compared with PSVT.

**Table 2:** Comparison of echocardiographic and laboratory parameters.

**Variables**	**PSVT (** **n** = 22**)**	**paAF (** **n** = 37**)**	**peAF (** **n** = 33**)**	**F**	**p** ** value**
Mean LAD (mm)	33.41 ± 4.60	38.14 ± 5.90^∗^	40.88 ± 5.97^∗^^#^	11.573	< 0.001
Mean LVEF (%)	63.68 ± 4.46	64.46 ± 5.28	60.79 ± 5.60^∗^^#^	4.597	0.013
Mean LVDD (mm)	47.00 ± 5.01	46.32 ± 2.79	48.48 ± 3.46^#^	3.117	0.049
Mean LV short-axis shortening rate (%)	34.09 ± 4.37	34.46 ± 2.85	32.33 ± 2.38^∗^^#^	4.345	0.016
Mean HCT (%)	39.82 ± 4.35	39.69 ± 3.48	42.23 ± 4.19^∗^^#^	4.184	0.018
Mean UA (mg/dL)	334.27 ± 124.39	317.01 ± 100.26	353.24 ± 87.76	1.092	0.340
Median TG (mmol/L)	1.26 (0.89, 2.01)	1.60 (1.31, 1.93)	1.30 (1.02, 1.60)	4.047	0.132
Median BNP (pg/mL)	102.00 (52.00, 287.75)	177.00 (119.00, 604.50)⁣^∗^	256.00 (98.00, 596.50)	8.376	0.015
Mean hs-CRP (mg/L)	13.94 ± 9.57	11.29 ± 6.77	12.97 ± 7.22	0.906	0.408
Mean Gal-3 (ng/mL)	12.04 ± 3.28	13.81 ± 2.56^∗^	15.92 ± 3.67^∗^^#^	10.228	< 0.001

Abbreviations: BNP, brain natriuretic peptide; Gal-3, galectin-3; HCT, hematocrit; hs-CRP, high-sensitivity C-reactive protein; LAD, left atrial diameter; LV, left ventricular; LVDD, end-diastolic left ventricular diameter; LVEF, left ventricular ejection fraction; paAF, paroxysmal atrial fibrillation; peAF, persistent atrial fibrillation; PSVT, paroxysmal supraventricular tachycardia; TGs, triglycerides; UA, uric acid.

⁣^∗^*p* < 0.05 compared with PSVT. ^#^*p* < 0.05 compared with paAF.

**Table 3:** Comparison of baseline variables among recurrence and nonrecurrence groups.

**Variables**	**Nonrecurrence (** **n** = 42**)**	**Recurrence (** **n** = 28**)**	**χ** ^2^/**t**/**Z**	**p** ** value**
Male, *n* (%)	29 (69.0)	19 (67.9)	0.011	0.916
Mean age (years)	65.38 ± 8.16	66.21 ± 9.24	−0.397	0.693
Mean BMI (kg/m^2^)	25.85 ± 3.33	25.30 ± 3.70	0.643	0.522
Mean SBP (mm Hg)	130.40 ± 15.69	138.43 ± 25.38	−1.493	0.143
Mean DBP (mm Hg)	83.33 ± 11.20	87.25 ± 10.86	−1.450	0.152
Smoking history, *n* (%)	17 (40.5)	12 (42.9)	0.039	0.843
Drinking history, *n* (%)	13 (31.0)	6 (21.4)	0.771	0.380
Hypertension, *n* (%)	31 (73.8)	15 (53.6)	3.054	0.081
DM, *n* (%)	2 (4.8)	4 (14.3)		0.209^a^
Lipid metabolism disorder, *n* (%)	3 (7.1)	5 (17.9)		0.252^a^
CHD, *n* (%)	20 (47.6)	14 (50.0)	0.038	0.845
OSAHS, *n* (%)	2 (4.8)	2 (7.1)		1.000^a^
Cerebrovascular events, *n* (%)	3 (7.1)	0 (0.0)		0.270
Mean LAD (mm)	37.60 ± 5.64	42.18 ± 5.68	−3.324	0.001
Mean LAV (mL/m^2^)	135.63 ± 23.01	158.95 ± 22.91	−4.161	< 0.001
Mean HCT (%)	40.20 ± 4.04	41.92 ± 3.80	−1.789	0.078
Mean UA (mg/dL)	333.86 ± 92.64	334.44 ± 101.72	−0.025	0.980
Median TG (mmol/L)	1.48 (1.19, 1.66)	1.43 (1.15, 2.01)	−0.378	0.705
Median BNP (pg/mL)	131.00 (103.75, 5561.25)	299.00 (165.75, 833.57)	−1.613	0.107
Mean hs-CRP (mg/L)	10.28 ± 5.99	14.79 ± 7.59	−2.638	0.011
Mean Gal-3 (ng/mL)	13.17 ± 2.41	17.25 ± 2.90	−6.377	< 0.001
Mean LVEF (%)	63.69 ± 4.29	61.29 ± 7.18	1.593	0.119
Mean LVDD (MM)	46.74 ± 2.79	48.25 ± 3.79	−1.811	0.077
Mean LV short-axis shortening rate (%)	34.02 ± 2.68	32.61 ± 2.88	2.100	0.039

Abbreviations: BMI, body mass index; BNP, brain natriuretic peptide; CHD, coronary heart disease; DBP, diastolic blood pressure; DM, diabetes mellitus; Gal-3, galectin-3; HCT, hematocrit; hs-CRP, high-sensitivity C-reactive protein; LA, left atrial; LAD, left atrial diameter; LAV, left atrial volume; LVDD, end-diastolic left ventricular diameter; LVEF, left ventricular ejection fraction; OSAHS, obstructive sleep apnea/hypopnea syndrome; paAF, paroxysmal atrial fibrillation; peAF, persistent atrial fibrillation; PSVT, paroxysmal supraventricular tachycardia; SBP, systolic blood pressure; TGs, triglycerides; UA, uric acid.

^a^Using Fisher's exact probability method.

**Table 4:** Multivariate logistic regression with recurrence as dependent variable.

**Variables**	**B**	**SE**	**Wald**	**p** ** value**	**OR**	**95% CI**	
						**Lower**	**Upper**
LAD	0.114	0.083	1.893	0.169	1.121	0.953	1.318
LAV	0.042	0.016	6.820	0.009	1.043	1.011	1.077
hs-CRP	0.162	0.070	5.314	0.021	1.176	1.025	1.349
Gal-3	0.703	0.212	10.961	0.001	2.019	1.332	3.06
Left ventricular short-axis shortening rate	−0.065	0.160	0.162	0.687	0.937	0.685	1.284

Abbreviations: CI, confidence interval; Gal-3, galectin-3; hs-CRP, high-sensitivity C-reactive protein; LA, left atrial; LAD, left atrial diameter; LAV, left atrial volume; LV, left ventricular; OR, odds ratio.

**Table 5:** Summary of baseline Gal-3 level comparison among recurrence and nonrecurrence groups.

**Study or subgroup**	**Country**	**With AF recurrence**		**Without AF recurrence**		**Multivariate analysis**
		**N**	**M** **e** **a** **n** ± **SD**	**N**	**M** **e** **a** **n** ± **SD**	
Wu et al. [14]	China	32	6.03 ± 2.12	18	4.28 ± 2.03	OR = 1.55, 95% CI: 1.038–2.318; *p* = 0.032
Kornej et al. [12]	Germany	36	7.09 ± 2.75	56	8.11 ± 3.13	—
Takemoto et al. [19]	United States	16	14.1 ± 2.8	39	11.9 ± 3.75	—
Clementy et al. [10]	France	55	16.1 ± 6.6	105	14.4 ± 5.6	HR = 1.07, 95% CI: 1.01–1.12; *p* = 0.02
Begg et al. [8]	United Kingdom	42	30.8 ± 75.4	50	24.5 ± 40.0	—
Lee et al. [3]	Republic of Korea	20	8.4 ± 2.1	55	6.9 ± 2.0	HR = 1.39, 95% CI: 1.14–1.70; *p* = 0.001
Ruan et al. [13]	China	35	14.97 ± 1.86	118	13.91 ± 1.88	HR = 1 : 28, 95% CI: 1.04–1.56; *p* = 0 : 02
Present study	China	28	17.25 ± 2.90	42	13.17 ± 2.41	OR = 2.019, 95% CI: 1.332–3.06; *p* = 0.001

Abbreviations: AF, atrial fibrillation; CI, confidence interval; Gal-3, galectin-3; HR, hazard ratio; OR, odds ratio.

## Data Availability

The data that support the findings of this study are available from the corresponding author upon reasonable request.
